# Combined use of two frailty tools in predicting mortality in older adults

**DOI:** 10.1038/s41598-022-19148-x

**Published:** 2022-09-03

**Authors:** Daiki Watanabe, Tsukasa Yoshida, Yosuke Yamada, Yuya Watanabe, Minoru Yamada, Hiroyuki Fujita, Motohiko Miyachi, Hidenori Arai, Misaka Kimura

**Affiliations:** 1 Faculty of Sport Sciences, Waseda University, 2-579-15 Mikajima, Tokorozawa, Saitama 359-1192 Japan; 2 National Institute of Health and Nutrition, National Institutes of Biomedical Innovation, Health and Nutrition, 1-23-1 Toyama, Shinjuku-ku, Tokyo 162-8636 Japan; 3 Institute for Active Health, Kyoto University of Advanced Science, 1-1 Nanjo Otani, Sogabe-cho, Kameoka, Kyoto 621-8555 Japan; 4 Senior Citizen’s Welfare Section, Kameoka City Government, 8 Nonogami, Yasumachi, Kameoka, Kyoto 621-8501 Japan; 5 Physical Fitness Research Institute, Meiji Yasuda Life Foundation of Health and Welfare, 150 Tobuki-machi, Hachioji, Tokyo 192-0001 Japan; 6 Faculty of Human Sciences, University of Tsukuba, 3-29-1 Otsuka, Bunkyo-ku, Tokyo 112-0012 Japan; 7 National Center for Geriatrics and Gerontology, 7-430 Morioka-cho, Obu, Aichi 474-8511 Japan; 8 Department of Nursing, Doshisha Women’s College of Liberal Arts, 97-1 Minamihokotate, Kodo, Kyotanabe, Kyoto 610-0395 Japan; 9 Laboratory of Applied Health Sciences, Kyoto Prefectural University of Medicine, 465 Kajii-cho, Kamigyo-ku, Kyoto 602-8566 Japan

**Keywords:** Health care, Risk factors

## Abstract

We aimed to verify the combined use of two frailty tools in predicting mortality in older adults. We used the data of 10,276 Japanese older adults (aged ≥ 65 years) who provided valid responses to two frailty assessment tools in a mail survey in Japan’s Kyoto‒Kameoka Prospective cohort study. Frailty status was categorized into four groups depending on the validated frailty screening index and Kihon Checklist, respectively: Non-frailty (*n* = 5960), Physical frailty (*n* = 223), Comprehensive frailty (*n* = 2211), and Combination (*n* = 1882) groups. Mortality data were collected between July 30, 2011, and November 30, 2016. We assessed the relationship between frailty status and all-cause mortality risk using multivariate Cox proportional hazards models. During a median follow-up of 5.3 years, we recorded 1257 deaths. After adjusting for confounders, the Combination group had the highest mortality risk compared with the other groups [Non-frailty: reference; Physical frailty: hazards ratio [HR], 0.99 (95% confidence interval [CI] 0.58 to 1.70); Comprehensive frailty: 1.91 (1.63 to 2.23); Combination: 2.85 (2.44 to 3.22)]. People who are positive for frailty in both instruments have a higher risk of death than those who are positive to one model.

## Introduction

Frailty is a condition wherein multiple physiological reserves deteriorates because of decreased homeostasis ability to cope with stress^1,2^. Frailty, which is the antonym of “fit,”^3,4^ indicates biological aging^5,6^; as such, it is a public health problem among older adults worldwide^7^. Therefore, to extend the healthy longevity of older adults, the effect of frailty—with a focus on biological aging—on prognosis should be assessed.

Frailty can be broadly assessed using two models: the phenotype model by Fried et al.^8^ and the deficit accumulation model by Mitnitski and Rockwood et al.^9,10^. The phenotype model mainly assesses physical characteristics^8^, whereas the deficit accumulation model reflects the accumulation of multiple factors, including social, cognitive factors, and physical aspects^9,10^ that may induce adverse events. Individuals identified as frail based on the definitions of these models may not necessarily match^11^. However, frailty is related to mortality^11–14^ and the risk of disability in older adults^11,13^, regardless of the type of model used.

There are more than 20 methods for assessing frailty^3^, and these methods differ from one study to another. Consequently, there were reports of inconsistencies in the classification of frailty and in the predictive capacity depending on the frailty assessment method used^11^. Therefore, it is possible that the prognoses of older adults who qualified frail as defined by the combined use of multiple frailty and single frailty assessment tool may differ^12^. Nevertheless, to the best of our knowledge, combining both tools for assessing frailty to determine mortality risk in Japanese older adults has not yet been investigated. Disability complications of frailty are reversible since individuals can return to a healthy state through appropriate lifestyle intervention^15,16^; therefore, it is essential to identify high-risk older adults for early detection and treatment of frailty. In this study, we aimed to conduct a community-based longitudinal cohort study among older adults to investigate the combined use of two validated frailty assessment tools in determining the risk of all-cause mortality. We hypothesized that the combined use of the two models would lead to a stronger association between frailty and mortality risk than the use of either model individually.

## Methods

### Study population and assessment of baseline characteristics

**Figure 1:**
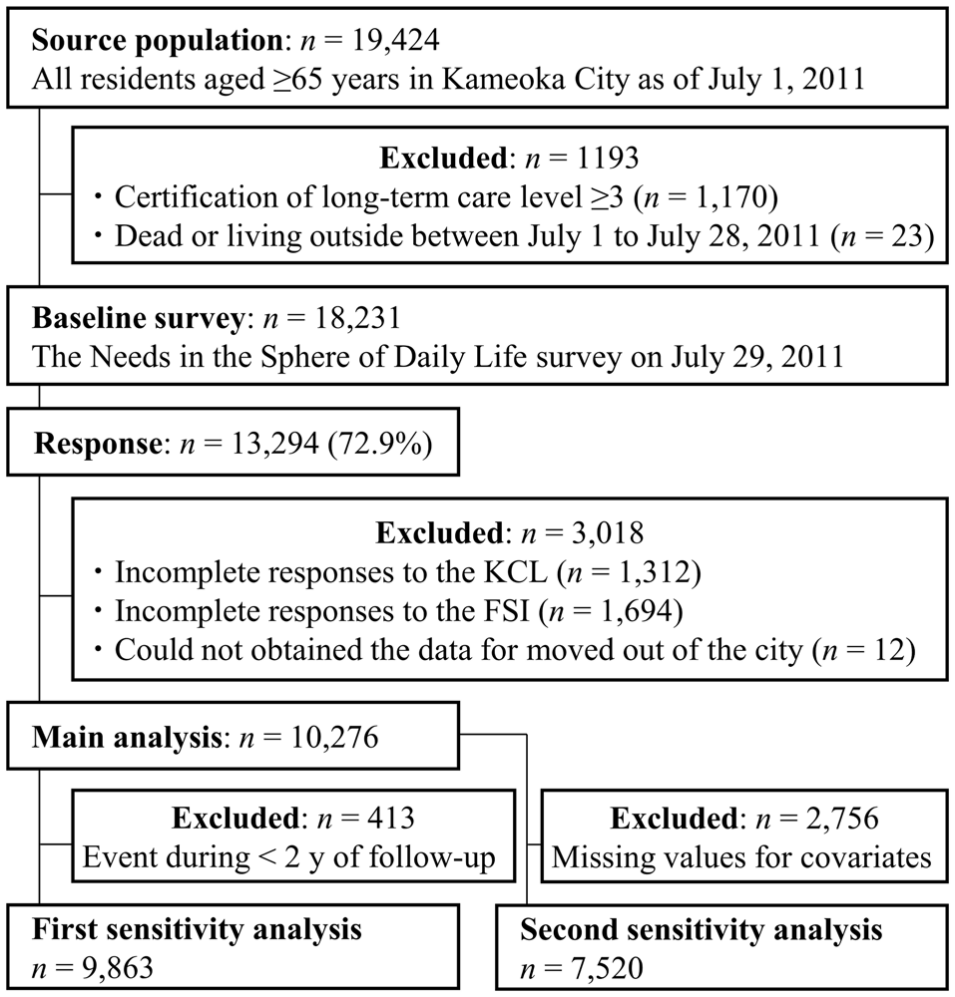
Participant flow diagram for the analysis of frailty status and mortality in Kyoto-Kameoka study. FSI, frailty screening index; KCL, Kihon Checklist.

The Kyoto-Kameoka study is a cohort study of older adults aged ≥ 65 years residing in Kameoka City, Kyoto Prefecture, Japan. The details of the Kyoto-Kameoka study have been published elsewhere^17–23^. A survey was conducted among the residents of Kameoka City, aged ≥ 65 years as of July 1, 2011. A municipal employee in charge of the survey selected qualified participants based on their name, sex, and date of birth, which were obtained from the Basic Resident Register maintained by Kameoka City Hall (Fig. 1). Finally, a total of 10,276 participants were included in this prospective study. We obtained informed consent from all participants upon receiving their responses to the mail-in survey. This study was conducted according to principles of the Declaration of Helsinki and all procedures involving research study participants were approved by the Research Ethics Committee of Kyoto Prefectural University of Medicine (RBMR-E-363), the National Institutes of Biomedical Innovation, Health and Nutrition (NIBIOHN-76-2), and Kyoto University of Advanced Science (No. 20-1). The reporting of this study conformed to the Strengthening the Reporting of Observational Studies in Epidemiology (STROBE)^24^.

### Definition of frailty

We used two frailty assessment tools, namely, self-administered frailty screening index (FSI), with five previously validated question items^20,25^, and self-administered Kihon Checklist (KCL), with 25 previously validated question items^20,26,27^ (Supplementary Tables 1 and 2). The FSI is based on the phenotype model, which mainly assesses frailty from the perspective of physical aspects (physical frailty); meanwhile, KCL is based on the accumulation of the deficit model, which assesses frailty from multidimensional perspectives (comprehensive frailty), including social, cognitive factors, and physical aspects. Physical frailty based on the FSI is defined as three or more of the five items^25^. Comprehensive frailty based on the KCL is defined as seven or more of the 25 items^20,26,27^. The seven subdomains included in the KCL (instrumental activities of daily living, physical function, nutritional status, oral function, social status, cognitive status, and depression) have been assessed in accordance with a previous study^28^.

### Outcomes

The survival status of the cohort participants during the follow-up period was assessed using the data from the Basic Resident Register maintained by Kameoka City Hall. These data were collected between July 30, 2011, and November 30, 2016. We performed censoring of residents who lost their official resident status, left the country, or moved to another municipality.

## Statistical analysis

The participants were assigned to one of the four groups: Non-frailty, when unqualified with either physical nor comprehensive frailty (*n* = 5960, 58.0%); Physical frailty, when unqualified with comprehensive frailty (*n* = 223, 2.2%); Comprehensive frailty, when unqualified with physical frailty (*n* = 2211, 21.5%); and Combination group, when qualified with both physical and comprehensive frailty (*n* = 1882, 18.3%).

Descriptive statistics for continuous variables are shown as the mean and standard deviation, and intergroup comparisons were performed using analysis of variance. Categorical variables are shown as number of people and percentage, and intergroup comparisons were performed using Pearson’s chi-square test. Where covariate information pertaining to the family structure (*n* = 675, 6.6%); socioeconomic status (*n* = 395, 3.8%); education (*n* = 1139, 11.0%); smoking status (*n* = 257, 2.5%); alcohol status (*n* = 230, 2.2%); sleep time (*n* = 536, 5.2%); and medications (*n* = 657, 6.4%) was missing, we imputed data using the five data sets created using multiple imputation by chained equation (MICE)^29^ package using R statistical software (process: m [number of imputed datasets] = 5; predictorMatrix = predmtx1; maxit [a scalar giving the number of iterations] = 50; meth [imputation method] = predictive mean matching [pmm], logreg, or polr 500 seed). The imputation method for continuous, binary, and ordered categorical variables used the pmm, logreg, and polr, respectively. To impute the missing data, the predictors used all Model 2 variables and variables that were associated with missingness^21^. The integrated parameter was calculated by arithmetic mean for the data sets. All missing values were assumed to be missing at random. It has been suggested that 5% missing data is the upper threshold for which multiple imputations provide a benefit in large data sets; if the missing values exceed 10%, it is stated that bias is likely in the analyses^30^. The details of the missing data pattern for covariates are shown in Supplementary Fig. 1. The number of complete cases without missing values was 7520 (73.2%). Furthermore, we compared the characteristics of the individuals who participated in this study with those who were excluded.

The absolute risk of all-cause mortality in each of the four groups in accordance with the degree of frailty is shown as the number of events per 1000 person-years. To adjust for confounding factors in the relationship between frailty and the risk of all-cause mortality, we used multivariate Cox proportional hazards analysis that included the baseline covariates. The Schoenfeld residuals test was performed to confirm the assumptions of the Cox proportional-hazard model. Proportional hazard conditions were assumed because the test did not reject the data (*p* = 0.173). We conducted multivariate analysis using the following two models: Model 1 was adjusted for age (continuous), sex (female or male), and population density (≥ 1000 or < 1000 people/km^2^), and Model 2 was further adjusted for the following factors in Model 1: living alone (yes or no), socioeconomic status (high or low), educational attainment (< 9, 10–12, or ≥ 13 years), smoking status (never smoker, past smoker, or current smoker), alcohol drinker (yes or no), sleep time (continuous), medication use (continuous), and number of chronic diseases (continuous). These adjustment factors were decided with reference to covariates used in previous studies that examined the association between frailty^11,31^, KCL^27^, or FSI^25^ and mortality or factors associated with prevalence of frailty^17,18,23^ before performing the statistical analysis. The results of these analyses are shown as hazards ratio (HR) and 95% confidence interval (CI), and HRs were calculated with the non-frailty group as the reference group. The interaction between physical and comprehensive frailty regarding the risk of mortality was assessed with relative excess risk due to interaction (RERI) as an additive interaction. We performed sensitivity analyses using the following two methods: (1) to remove the possibility of a causal reversal relationship, we excluded mortality events (males: 244, females: 169) that were recorded during the first two years of the follow-up survey^19^; (2) we performed the same analyses using complete case datasets that included no missing values^32^. We examined the relationship between the subdomains included in the FSI and KCL, which were used for assessing frailty, and the risk of all-cause mortality in the same way. In addition, we constructed adjusted Kaplan–Meier survival curves using the inverse probability weighting including the variables of adjustment Model 2^33^.

To assess the curvature of the relationship between the FSI and KCL scores and the risk of all-cause mortality, we used the restricted cubic spline model with three data points (5th, 50th, and 95th percentiles) based on the score distributions for these two assessments^22,23^. The results are shown as HR and 95% CI, with HR being calculated based on FSI and KCL scores of 0 points (no frailty).

The significance for all statistical analyses was set at < 5% on both sides. All statistical analyses were performed using STATA MP version 15.0 (StataCorp LP, College Station, TX, USA), and/or R software 3.4.3 (R Core Team, Vienna, Austria).

## Results

### Population and exposure characteristics

The characteristics of the participants in each of the four or two groups corresponding to the frailty status are shown in Table 1 and Supplementary Table 3. When compared to the Non-frailty group, the Combination group participants were older, predominantly women, and had a lower level of educational attainment. The prevalence rates of Physical and Comprehensive frailty were 20.5% and 39.8%, respectively (Supplementary Table 4). Participants in the Combination group had a higher prevalence of the subdomains of KCL and FSI than individuals with only one of those conditions (Supplementary Table 5). Meanwhile, the individuals excluded from this study were older, and the majority were women compared to those included in this study (Supplementary Table 6).

**Table 1:** Baseline characteristics of participants by frailty status.

	Total (*n* = 10,276)		Frailty status								*p*-value
			Non-frailty (*n* = 5960)		Physical frailty (*n* = 223)		Comprehensive frailty (*n* = 2211)		Combinations (*n* = 1882)		
Age [years]^a^	73.9	(6.8)	71.8	(5.3)	72.0	(5.4)	76.0	(7.3)	78.5	(7.7)	< 0.001
Women [*n* (%)]^b^	5580	(54.3)	3041	(51.0)	121	(54.3)	1289	(58.3)	1129	(60.0)	< 0.001
PD ≥ 1000 people/km^2^ [*n* (%)]^b^	4633	(45.1)	2772	(46.5)	94	(42.2)	968	(43.8)	799	(42.5)	0.006
Living alone [*n* (%)]^b^	1287	(12.5)	691	(11.6)	23	(10.3)	272	(12.3)	301	(16.0)	< 0.001
HSES [*n* (%)]^b^	3339	(32.5)	2200	(36.9)	73	(32.7)	619	(28.0)	447	(23.8)	< 0.001
Education ≥ 13 y [*n* (%)]^b^	2103	(20.5)	1414	(23.7)	59	(26.5)	339	(15.3)	291	(15.5)	< 0.001
Current smoker [*n* (%)]^b^	1126	(11.0)	692	(11.6)	25	(11.2)	209	(9.5)	200	(10.6)	0.002
Alcohol drinker [*n* (%)]^b^	6482	(63.1)	4102	(68.8)	156	(70.0)	1283	(58.0)	941	(50.0)	< 0.001
Sleep time [min/day]^a^	412	(94)	403	(72)	400	(81)	420	(103)	431	(135)	< 0.001
No medication [*n* (%)]^b^	2104	(20.5)	1524	(25.6)	39	(17.5)	342	(15.5)	199	(10.6)	< 0.001
Hypertension [*n* (%)]^b^	3894	(37.9)	2161	(36.3)	96	(43.0)	895	(40.5)	742	(39.4)	0.001
Stroke [*n* (%)]^b^	476	(4.6)	138	(2.3)	5	(2.2)	175	(7.9)	158	(8.4)	< 0.001
Heart disease [*n* (%)]^b^	1275	(12.4)	524	(8.8)	32	(14.3)	329	(14.9)	390	(20.7)	0.006
Diabetes [*n* (%)]^b^	1108	(10.8)	546	(9.2)	24	(10.8)	274	(12.4)	264	(14.0)	0.087
Hyperlipidaemia [*n* (%)]^b^	924	(9.0)	580	(9.7)	21	(9.4)	181	(8.2)	142	(7.5)	0.015
Digestive disease [*n* (%)]^b^	499	(4.9)	167	(2.8)	18	(8.1)	132	(6.0)	182	(9.7)	< 0.001
Respiratory disease [*n* (%)]^b^	824	(8.0)	357	(6.0)	28	(12.6)	200	(9.0)	239	(12.7)	< 0.001
Urological diseases [*n* (%)]^b^	646	(6.3)	265	(4.4)	14	(6.3)	155	(7.0)	212	(11.3)	< 0.001
Cancer [*n* (%)]^b^	367	(3.6)	145	(2.4)	10	(4.5)	86	(3.9)	126	(6.7)	< 0.001
No. of chronic diseases^a,c^	0.9	(1.0)	0.8	(0.9)	1.1	(1.0)	1.1	(1.0)	1.3	(1.2)	< 0.001

Data for participants with missing values were imputed by multiple imputation: family structure (*n* = 675); socioeconomic status (*n* = 395); education (*n* = 1139); smoking status (*n* = 257); alcohol status (*n* = 230); sleep time (*n* = 536); medications (*n* = 657).

*HSES* high socioeconomic status, *PD* population density.

^a^Continuous variables were shown in terms of mean with standard deviation and were analysed using variance analysis.

^b^Category variables were shown in terms of the number of cases with percentage and were analysed using the Pearson's Chi-square test.

^c^From the data obtained on disease status (including the presence of hypertension, stroke, heart disease, diabetes, hyperlipidaemia, digestive disease, respiratory disease, urological diseases, and cancer), the comorbidity scores were summed to obtain a total score ranging from 0 (no comorbidity) to 9 (poor status)^19^.

### Combined use of two frailty concept models in mortality

**Figure 2:**
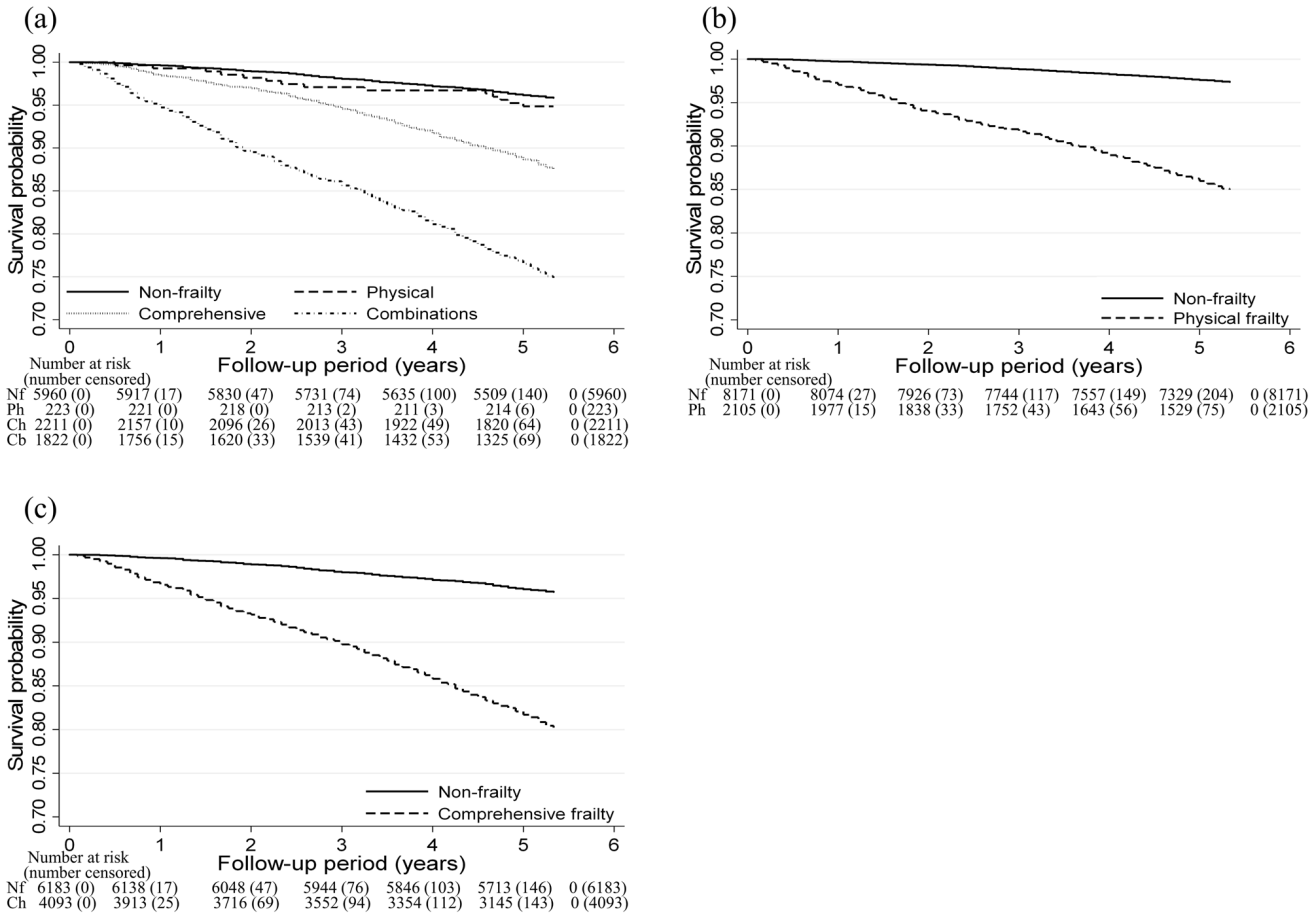
Multivariate adjusted Kaplan–Meier survival curves using inverse probability weighting for all-cause mortality according to frailty status among older adults. (**a**) Four groups stratified by frailty screening index (FSI) and Kihon Checklist (KCL); (**b**) two groups stratified by FSI; (**c**) two groups stratified by KCL. Nf, non-frailty; Ph, physical frailty; Ch, comprehensive frailty; Cb, combinations. The adjustment factors are age, sex, population density, family structure, economic status, educational attainment, smoking status, alcohol consumption status, sleep time, medication use, and number of chronic diseases.

The relationships between frailty status and the risk of all-cause mortality are shown in Fig. 2 and Table 2. The median follow-up period was 5.3 years (50,984 person-years). During the follow-up period, 1257 individuals (12.2%) died. Even after adjusting for confounding factors, the Combination group had the highest risk for all-cause mortality relative to the other groups [Non-frailty group: reference; Physical frailty: HR, 0.99 (95% CI 0.58–1.70); Comprehensive frailty: HR, 1.91 (95% CI 1.63–2.23); Combination: HR, 2.85 (95% CI 2.44–3.22), *p* < 0.001]. The interaction between Physical and Comprehensive frailty accounted for 51.3% relative excess risk of mortality in the Combinations group. Several of the sensitivity analyses also yielded the same results but the sensitivity analysis after excluding participants with an event in the first two years of follow-up was not significant for the RERI using a categorical frail/non-frail variable for KCL and FSI (Supplementary Tables 7 and 8). In addition, these findings were similar to the results using frailty defined by FSI of ≥ 2 points (Supplementary Table 9). The same results were observed even when the samples were stratified by age or sex, with more pronounced results obtained for men and individuals aged ≥ 75 years (Supplementary Tables 10 and 11). Moreover, there was a relationship between all subdomains included in the FSI and KCL and the risk of all-cause mortality (Supplementary Table 12).

**Table 2:** Hazard ratios for frailty status and all-cause mortality calculated using multivariate Cox proportional hazards analysis.

	*n*	Event	PY	Event/1000 PY		Model 1^a^		Model 2^b^	
				Rate	95%CI	HR	95%CI	HR	95%CI
**FSI × KCL**									
Non-frailty	5960	346	30,677	11.3	(10.2 to 12.5)	1.00	(Ref)	1.00	(Ref)
Physical frailty	223	14	1146	12.2	(7.2 to 20.6)	1.05	(0.62 to 1.80)	0.99	(0.58 to 1.70)
Comprehensive frailty	2211	367	10,792	34.0	(30.7 to 37.7)	2.02	(1.74 to 2.36)	1.91	(1.63 to 2.23)
Combinations	1882	530	8368	63.3	(58.2 to 69.0)	3.16	(2.72 to 3.66)	2.85	(2.44 to 3.22)
*Interaction*									
RERI^c^				28.4	(22.1 to 34.7)	1.08	(0.45 to 1.71)	0.95	(0.33 to 1.57)
RERI (%)				54.6		50.0		51.3	
**FSI**									
Non-frailty	8171	713	41,469	17.2	(16.0 to 18.5)	1.00	(Ref)	1.00	(Ref)
Physical frailty	2105	544	9514	57.2	(52.6 to 62.2)	2.07	(1.84 to 2.34)	1.89	(1.67 to 2.13)
**KCL**									
Non-frailty	6183	360	31,823	11.3	(10.2 to 12.5)	1.00	(Ref)	1.00	(Ref)
Comprehensive frailty	4093	897	19,161	46.8	(43.8 to 50.0)	2.51	(2.20 to 2.86)	2.30	(2.00 to 2.63)

*CI* confidence interval, *FSI* frailty screening index, *HR* hazard ratio, *KCL* kihon checklist, *RERI* relative excess risk due to interaction, *PY* person-years.

^a^Model 1: Adjusted for age, sex, and population density.

^b^Model 2: In addition to the factors listed in Model 1, adjusted for family structure, economic status, educational attainment, smoking status, alcohol consumption status, sleep time, medication use, and number of chronic diseases.

^c^We estimated that *p* < 0.05 when the 95% CI of the RERI exceeded 0, and *p* ≥ 0.05 when the 95% CI of the RERI did not exceed 0.

### Dose-response relationships

**Figure 3:**
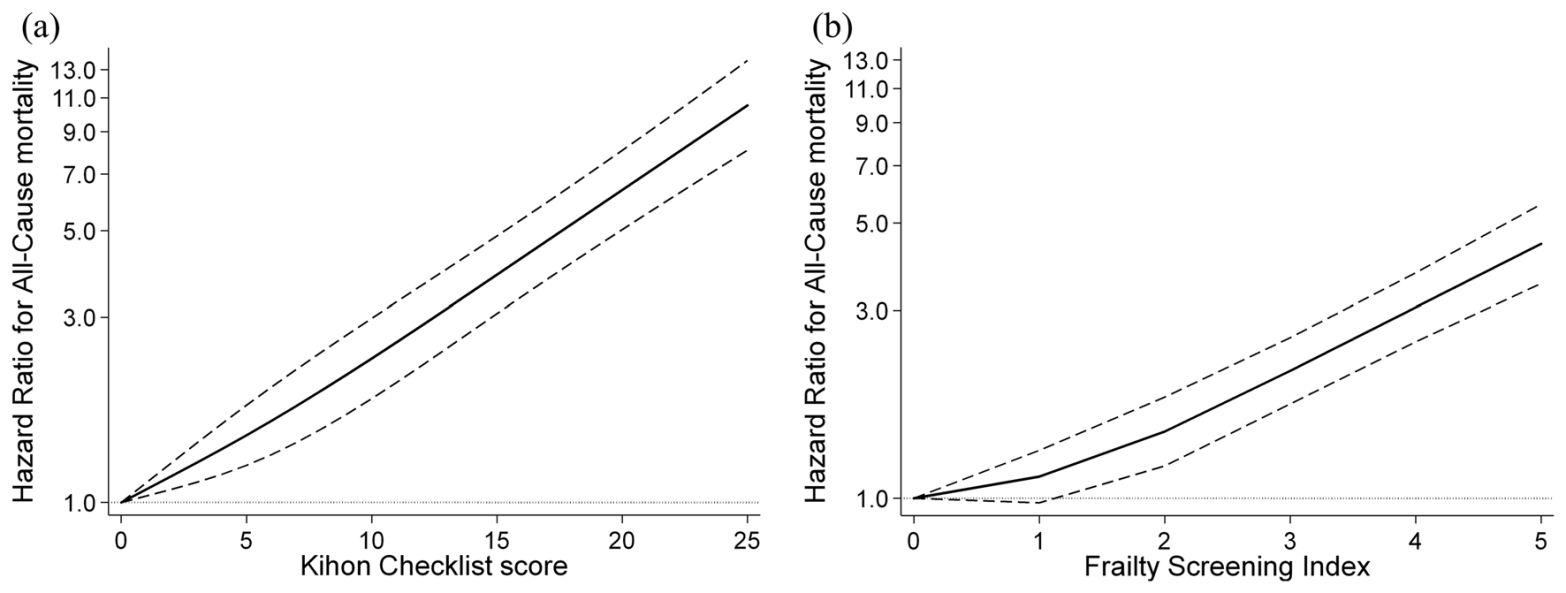
Restricted cubic spline regression model between KCL (**a**) and FSI (**b**) score and risk of all-cause mortality. The Kihon Checklist (KCL) and Frailty Screening Index (FSI) received a point by every problem with activity or function, and the higher the total score, the greater the difficulty in daily functioning (high frailty). Solid lines represent hazard ratios, and dashed lines represent 95% confidence intervals (CI), and the hazard ratio based on 0 point of both KCL and FSI as reference was calculated. We estimated that *p* < 0.05 when the 95% CI of the hazard ratio exceeded 1.00, and *p* ≥ 0.05 when the 95% CI of the hazard ratio did not exceed 1.00. The adjustment factors are age, sex, population density, family structure, economic status, educational attainment, smoking status, alcohol consumption status, sleep time, medication use, and number of chronic diseases.

We assessed the curvature relationship of the FSI and KCL scores with the risk of mortality using the restricted cubic spline model (Fig. 3). Even after adjusting for baseline confounding factors, there was a strong log linear relationship between both scores and the risk of all-cause mortality in a dose–response-dependent manner (did not show a linear relationship for FSI ≤ 2 points). Multivariate-adjusted HR (95% CI) of all-cause mortality for each one-point increment was 1.36 (1.29–1.42) for FSI and 1.10 (1.09–1.11) for KCL.

## Discussion

### Main findings

In this population-based cohort study of older adults, we examined the combined use of two verified frailty assessment tools in assessing the risk of mortality. After adjusting for confounders, we found that the Combination group had the strongest association with the risk of all-cause mortality compared with the other groups. To the best of our knowledge, this is the first study to verify the combined use of two frailty tools to assess the relationship between frailty status and mortality risk in Japanese individuals. Our results suggest that frailty assessed by the combined use of multiple frailty assessment tools may have a higher predictive ability for prognosis than frailty assessed using any single tool.

### Prevalence of physical and comprehensive frailty

Our results showed that the prevalence of Comprehensive frailty (39.8%) was higher than that of Physical frailty (20.5%), which is consistent with the findings of previous studies^21,23^. These prevalence of frailty is relatively higher than what was reported in other well-established cohorts^34^. This could be attributed to the fact that the prevalence of frailty assessed using KCL was higher than those assessed using commonly used screening instruments^26^. Comprehensive frailty, as defined using the KCL, included multiple components such as cognitive, social, and other factors. In contrast, physical frailty, as defined using the FSI, focuses mainly on the physical aspects. Thus, consideration must be given to the possibility that the pathologies resulting from comprehensive and physical frailty may differ^35^. However, approximately 90% of the people with physical frailty in our study had comprehensive frailty. Therefore, it is important to assess the prognosis of those with both physical and comprehensive frailty and those with a single type of frailty.

### Main outcomes

A previous study that followed-up 4721 older adults aged ≥ 65 years for four years found that individuals who were deemed to be frail based on both phenotype and deficit accumulation model had the highest risk of mortality^12^. In our study, we found the same results when we used the data of more than 10,000 individuals (50,984 person-years), which is more than double the size of that of the previous study. Previous studies that compared comprehensive frailty, considering multiple factors, and physical frailty, which focuses on physical factors only, reported that comprehensive frailty had higher accuracy in predicting the risk of mortality^12,14^. This could be due to the fact that comprehensive frailty indices, which assess frailty using a multi-faceted model have a linear positive correlation with age, and thus reflect biological aging^5,6^. Furthermore, middle-aged and older adults with both sarcopenia and frailty have a higher rate of all-cause mortality^36,37^ and a higher risk of cardiovascular and respiratory diseases^36^ than individuals with only one of those conditions. Findings of previous studies corroborates our results. Therefore, frailty defined through the combined use of two frailty tools may better identify the high-risk group than only either of the assessment models. However, a previous study concluded that combining different screening instruments does not improve predictive power of dependency, mortality, and hospitalization^38^. The study included only pre-frail and frail patients and did not include non-frail individuals. Our study population consisted of both frail and non-frail individuals as our sample was from the general population. We recommend further exploration of other combinations of instruments among other study populations because these differences might have affected the results. In addition, these detailed mechanisms need to be elucidated through interventional and basic research^35^. The data shown in our study may be helpful in identifying those with poorer prognoses of older adults.

### Dose-response relationships

Furthermore, we found a log linear positive dose–response relationship between the mortality risk and the FSI and KCL scores, which are used to assess frailty (did not show a linear relationship for FSI ≤ 2 points). This observation concurs with the phenotypic definition of frailty^12^. No previous studies have investigated the dose–response relationship between FSI/KCL scores and mortality risk^25,27^. Considering that the frailty is understood to exist on a spectrum from fit to frail^3,4^, our results appear to appreciate this with the identification of a dose-response with each of the two frailty instruments, whereas the use of sequential frailty categorization to define frailty populations will lead to loss of information value from each of these tools that are under and over this categorization threshold. Therefore, it is likely to be useful in the early prediction of the prognosis of older adults by spectrum from fit to frail using these indices with continuous variables.

### Strengths and limitations

The strength of this study is that we investigated the relationship of the risk of all-cause mortality with physical and comprehensive frailty defined using two validated assessment tools in a large-scale cohort study of community-dwelling older adults. Nevertheless, this study had several methodological limitations. First, although we used the validated frailty assessment tools, there may have been a self-reporting bias since our survey was based on a self-administered questionnaire. Two tools developed in the Japanese population were applied in defining frailty. This may be limited in its generalizability to other populations. There is need to re-evaluate using the most commonly used instruments for frailty assessment (Fried’s Phenotype model and the Rockwood’s Frailty Index)^3^ because our results may vary depending on the tool used to determine frailty. Although frailty status has an intermediate pre-frailty category, frailty assessments were dichotomized as frail or non-frail in this study. The dichotomization of frailty status is an over-simplification of the concept of frailty and the results may lead to misclassification of frailty status due to the dependence on the cut-off value used to determine frailty. This kind of misclassification in the exposure assessments might have weakened the relationship between mortality risk and frailty. Despite this, our study confirmed the relationship between mortality risk and frailty. Second, the observation period in our study was relatively short. This may have affected the relationship between frailty and mortality risk. In addition, because we could not obtain data on the cause of death, we did not examine whether frailty might have been linked to various causes of death. Furthermore, there is the possibility of selection bias due to different participant characteristics such as age and sex between participants included and excluded from this study. Third, physical frailty, as assessed using the FSI, is not perfectly consistent with the phenotype model advocated by Fried et al.^8^. The phenotype model includes grip strength (weakness)^8^, but because this index requires actual measurements, FSI utilizes cognitive function instead of referencing several frailty assessment questionnaires^25^. However, we confirmed the good predictivity of KCL and FSI against frailty defined by the revised Japanese version of the Cardiovascular Health Study criteria according to the Fried phenotype model in the sub-cohort of Kyoto-Kameoka study, which measured grip strength and gait speed^20^. In addition, we have previously reported that FSI can predict disability in older Japanese adults^25^. Fourth, although we found nearly no difference in the overall results of our observational study even after adjusting for several participant characteristics of participants and lifestyle-related factors, there may still be remaining confounding factors linked to frailty and the risk of all-cause mortality. Finally, although we imputed data using the multiple imputation method for missing data, the choices of the imputation methods, predictors, and the number of imputations (number of imputed datasets), among others, could have a significant impact on the quality of imputation. These limitations may make it difficult to generalize the study results.

### Perspective

In recent years, there has been a paradigm shift from a focus on individual diseases to a perception that multiple chronic diseases have common risk factors^39^ and that the simultaneous presence of multiple pathological conditions has a powerful influence over people’s health trajectory, disabilities, and the complexity of the care they require^40^. Frailty occurs because of complex interactions between various factors^41^. Thus, assessing frailty from a multifaceted perspective through the use of multiple frailty assessment indices may allow more sensitive identification of older adults with poor prognoses, including mortality. Considering that the degree of frailty observed across most adult age groups increased in the United States during 1999–2018^42^, our results may provide useful clues that can be used to identify high-risk older adults.

## Conclusion

Our results suggested that the combined use of tools to assess frailty was more strongly linked to the risk of mortality among older adults. These results suggest that assessment of frailty from a multifaceted approach using multiple frailty assessment indices may allow high-sensitive identification of older adults with poor prognoses and increased risk of mortality.

## Supplementary Information

Supplementary Information.

## Data Availability

The datasets used and/or analysed during the current study available from the corresponding author (d2watanabe@nibiohn.go.jp), TY (t-yoshida@nibiohn.go.jp), and YY (yamaday@nibiohn.go.jp) on reasonable request.
