# Supplementary material for: Combined use of two frailty tools in predicting mortality in older adults
Source: Sci Rep. 2022 Sep 3;12:15042. doi: 10.1038/s41598-022-19148-x (PMC9440890; doi:10.1038/s41598-022-19148-x)
Supplement: Supplementary file 1 — Supplementary Information. [file 41598_2022_19148_MOESM1_ESM.docx]

Supporting information

“Combined use of two frailty tools in predicting mortality in older adults”

Authors: Daiki Watanabe, Tsukasa Yoshida, Yosuke Yamada, Yuya Watanabe, Minoru Yamada, Hiroyuki Fujita, Motohiko Miyachi, Hidenori Arai, and Misaka Kimura

**SUPPLEMENTARY TABLES**

**Supplementary Table 1.** English translation of the Kihon Checklist

**Supplementary Table 2.** English translation of the frailty screening index

**Supplementary Table 3.** Comparison of baseline participant characteristics by frailty status defined by frailty screening index or Kihon Checklist

**Supplementary Table 4.** Distribution of frailty screening index and Kihon Checklist scores

**Supplementary Table 5.** Prevalence rate of the subdomains KCL and FSI by frailty status

**Supplementary Table 6.** Characteristics of the included and excluded participants

**Supplementary Table 7.** Results of sensitivity analysis for the relationship between frailty status and all-cause mortality after excluding participants with an event in the first two years of follow-up

**Supplementary Table 8.** Results of sensitivity analysis for the relationship between frailty status and all-cause mortality using complete case data

**Supplementary Table 9.** Results of sensitivity analysis for the relationship between frailty status and all-cause mortality using frailty defined by ≥ 2 point of frailty screening index

**Supplementary Table 10.** Hazard ratios for frailty status and all-cause mortality calculated using age-stratified multivariate Cox proportional hazards analysis

**Supplementary Table 11.** Hazard ratios for frailty status and all-cause mortality calculated using sex-stratified multivariate Cox proportional hazards analysis

**Supplementary Table 12.** Multivariate Cox proportional hazards analysis for the subdomains KCL and FSI and all-cause mortality

**Supplementary Figure 1.** Missing data pattern for covariates in this study

Supplementary Table 1. English translation of the Kihon Checklist

| No | Questions | Answer | |
| --- | --- | --- | --- |
| 1 | Do you go out by bus or train by yourself? | 0. YES | 1. NO |
| 2 | Do you go shopping to buy daily necessities by yourself? | 0. YES | 1. NO |
| 3 | Do you manage your own deposits and savings at the bank? | 0. YES | 1. NO |
| 4 | Do you sometimes visit your friends? | 0. YES | 1. NO |
| 5 | Do you turn to your family or friends for advice? | 0. YES | 1. NO |
| 6 | Do you normally climb stairs without using handrail or wall for support? | 0. YES | 1. NO |
| 7 | Do you normally stand up from a chair without any aids? | 0. YES | 1. NO |
| 8 | Do you normally walk continuously for 15 minutes? | 0. YES | 1. NO |
| 9 | Have you experienced a fall in the past year? | 1. YES | 0. NO |
| 10 | Do you have a fear of falling while walking? | 1. YES | 0. NO |
| 11 | Have you lost 2 kg or more in the past 6 months? | 1. YES | 0. NO |
| 12 | Height: cm, weight: kg, BMI: kg/m^2^ If body mass index is less than 18.5, this item is scored. | 1. YES | 0. NO |
| 13 | Do you have any difficulties eating tough foods compared to 6 months ago? | 1. YES | 0. NO |
| 14 | Have you choked on your tea or soup recently? | 1. YES | 0. NO |
| 15 | Do you often experience having a dry mouth? | 1. YES | 0. NO |
| 16 | Do you go out at least once a week? | 0. YES | 1. NO |
| 17 | Do you go out less frequently compared to last year? | 1. YES | 0. NO |
| 18 | Do your family or your friends point out your memory loss? e.g.“You ask the same question over and over again.” | 1. YES | 0. NO |
| 19 | Do you make a call by looking up phone numbers? | 0. YES | 1. NO |
| 20 | Do you find yourself not knowing today’s date? | 1. YES | 0. NO |
| 21 | In the last 2 weeks have you felt a lack of fulfilment in your daily life? | 1. YES | 0. NO |
| 22 | In the last 2 weeks have you felt a lack of joy when doing the things you used to enjoy? | 1. YES | 0. NO |
| 23 | In the last 2 weeks have you felt difficulty in doing what you could do easily before? | 1. YES | 0. NO |
| 24 | In the last 2 weeks have you felt helpless? | 1. YES | 0. NO |
| 25 | In the last 2 weeks have you felt tired without a reason? | 1. YES | 0. NO |

Supplementary Table 2. English translation of the frailty screening index

| No | Questions | Answer | |
| --- | --- | --- | --- |
| 1 | Have you lost 2 kg or more in the past 6 months? | 1. YES | 0. NO |
| 2 | Do you think you walk slower than before? | 1. YES | 0. NO |
| 3 | Do you go for a walk for your health at least once a week? | 1. YES | 0. NO |
| 4 | Can you recall what happened 5 minutes ago? | 0. YES | 1. NO |
| 5 | In the past 2 weeks, have you felt tired without a reason? | 1. YES | 0. NO |

Supplementary Table 3. Comparison of baseline participant characteristics by frailty status defined by frailty screening index or Kihon Checklist ^*^

|  | FSI | | | | *p*-value |  | KCL | | | | *p*-value |
| --- | --- | --- | --- | --- | --- | --- | --- | --- | --- | --- | --- |
|  | Non-frailty (*n* = 8171) | | Physical frailty (*n* = 2105) | |  |  | Non-frailty (*n* = 6183) | | Comprehensive frailty (*n* = 4093) | |  |
| Age [years] ^†^ | 72.9 | (6.2) | 77.8 | (7.7) | <0.001 |  | 71.8 | (5.3) | 77.2 | (7.6) | <0.001 |
| Women [*n* (%)] ^‡^ | 4330 | (53.0) | 1250 | (59.4) | <0.001 |  | 3162 | (51.1) | 2418 | (59.1) | <0.001 |
| PD ≥1000 people/km^2^ [*n* (%)] ^‡^ | 3740 | (45.8) | 893 | (42.4) | 0.006 |  | 2866 | (46.4) | 1767 | (43.2) | 0.002 |
| Living alone [*n* (%)] ^‡^ | 963 | (11.8) | 324 | (15.4) | <0.001 |  | 714 | (11.5) | 573 | (14.0) | <0.001 |
| HSES [*n* (%)] ^‡^ | 2819 | (34.5) | 520 | (24.7) | <0.001 |  | 2273 | (36.8) | 1066 | (26.0) | <0.001 |
| Education ≥13 y [*n* (%)] ^‡^ | 1753 | (21.5) | 350 | (16.6) | <0.001 |  | 1473 | (23.8) | 630 | (15.4) | <0.001 |
| Current smoker [*n* (%)] ^‡^ | 901 | (11.0) | 225 | (10.7) | 0.311 |  | 717 | (11.6) | 409 | (10.0) | <0.001 |
| Alcohol drinker [*n* (%)] ^‡^ | 5385 | (65.9) | 1097 | (52.1) | <0.001 |  | 4258 | (68.9) | 2224 | (54.3) | <0.001 |
| Sleep time [min] ^†^ | 407 | (82) | 428 | (130) | <0.001 |  | 403 | (72) | 425 | (119) | <0.001 |
| No medication [*n* (%)] ^‡^ | 1866 | (22.8) | 238 | (11.3) | <0.001 |  | 1563 | (25.3) | 541 | (13.2) | <0.001 |
| Hypertension [*n* (%)] ^‡^ | 3056 | (37.4) | 838 | (39.8) | 0.042 |  | 2257 | (36.5) | 1637 | (40.0) | <0.001 |
| Stroke [*n* (%)] ^‡^ | 313 | (3.8) | 163 | (7.7) | <0.001 |  | 143 | (2.3) | 333 | (8.1) | <0.001 |
| Heart disease [*n* (%)] ^‡^ | 853 | (10.4) | 422 | (20.0) | <0.001 |  | 556 | (9.0) | 719 | (17.6) | <0.001 |
| Diabetes [*n* (%)] ^‡^ | 820 | (10.0) | 288 | (13.7) | <0.001 |  | 570 | (9.2) | 538 | (13.1) | <0.001 |
| Hyperlipidemia [*n* (%)] ^‡^ | 761 | (9.3) | 163 | (7.7) | 0.025 |  | 601 | (9.7) | 323 | (7.9) | 0.002 |
| Digestive disease [*n* (%)] ^‡^ | 299 | (3.7) | 200 | (9.5) | <0.001 |  | 185 | (3.0) | 314 | (7.7) | <0.001 |
| Respiratory disease [*n* (%)] ^‡^ | 557 | (6.8) | 267 | (12.7) | <0.001 |  | 385 | (6.2) | 439 | (10.7) | <0.001 |
| Urological diseases [*n* (%)] ^‡^ | 420 | (5.1) | 226 | (10.7) | <0.001 |  | 279 | (4.5) | 367 | (9.0) | <0.001 |
| Cancer [*n* (%)] ^‡^ | 231 | (2.8) | 136 | (6.5) | <0.001 |  | 155 | (2.5) | 212 | (5.2) | <0.001 |
| No. of chronic diseases ^†,§^ | 0.9 | (0.9) | 1.3 | (1.2) | <0.001 |  | 0.8 | (0.9) | 1.2 | (1.1) | <0.001 |

HSES, high socioeconomic status; PD, population density

^*^ Data for participants with missing values were imputed by multiple imputation: family structure (*n* = 675); socioeconomic status (*n* = 395); education (*n* = 1139); smoking status (*n* = 257); alcohol status (*n* = 230); sleep time (*n* = 536); medications (*n* = 657).

^†^ Continuous variables were shown in terms of mean with standard deviation and were analyzed using variance analysis.

^‡^ Category variables were shown in terms of the number of cases with percentage and were analyzed using the Pearson's Chi-square test.

^§^ From the data obtained on disease status (including the presence of hypertension, stroke, heart disease, diabetes, hyperlipidemia, digestive disease, respiratory disease, urological diseases, and cancer), the individual scores were summed to obtain a total score ranging from 0 (no comorbidity) to 9 (poor status).

Supplementary Table 4. Distribution of frailty screening index and Kihon Checklist scores

| **KCL scores** | **FSI scores** | | | | | | **Total** |
| --- | --- | --- | --- | --- | --- | --- | --- |
|  | 0 | 1 | 2 | 3 | 4 | 5 |  |
| 0 | 511 | 196 | 15 | 1 | 0 | 0 | 723 |
| 1 | 631 | 419 | 69 | 6 | 0 | 0 | 1125 |
| 2 | 467 | 484 | 134 | 15 | 1 | 0 | 1101 |
| 3 | 314 | 535 | 170 | 23 | 2 | 1 | 1045 |
| 4 | 226 | 424 | 220 | 48 | 5 | 1 | 924 |
| 5 | 94 | 357 | 196 | 49 | 7 | 1 | 704 |
| 6 | 51 | 252 | 195 | 55 | 8 | 0 | 561 |
| 7 | 39 | 234 | 192 | 75 | 5 | 0 | 545 |
| 8 | 30 | 170 | 193 | 92 | 14 | 0 | 499 |
| 9 | 20 | 113 | 165 | 117 | 18 | 0 | 433 |
| 10 | 13 | 80 | 158 | 120 | 26 | 1 | 398 |
| 11 | 4 | 64 | 122 | 106 | 36 | 5 | 337 |
| 12 | 5 | 47 | 93 | 92 | 23 | 2 | 262 |
| 13 | 1 | 29 | 98 | 93 | 33 | 0 | 254 |
| 14 | 2 | 26 | 93 | 99 | 43 | 3 | 266 |
| 15 | 2 | 23 | 51 | 87 | 33 | 1 | 197 |
| 16 | 0 | 11 | 46 | 80 | 32 | 5 | 174 |
| 17 | 0 | 8 | 38 | 81 | 46 | 3 | 176 |
| 18 | 0 | 3 | 18 | 63 | 51 | 6 | 141 |
| 19 | 0 | 2 | 8 | 56 | 36 | 8 | 110 |
| 20 | 0 | 0 | 5 | 38 | 47 | 13 | 103 |
| 21 | 0 | 0 | 4 | 28 | 39 | 8 | 79 |
| 22 | 0 | 0 | 1 | 9 | 35 | 10 | 55 |
| 23 | 0 | 0 | 0 | 12 | 13 | 12 | 37 |
| 24 | 0 | 0 | 0 | 1 | 17 | 4 | 22 |
| 25 | 0 | 0 | 0 | 0 | 1 | 4 | 5 |
| **Total** | 2410 | 3477 | 2284 | 1446 | 571 | 88 | 10276 |

FSI, frailty screening index; KCL, Kihon Checklist

All value were shown in terms of the number of cases.

The prevalence of frailty defined by FSI (≥3 out of 5 points) and the KCL (≥7 out of 25 points) in the Kyoto–Kameoka Study were 20.5% (2105 people) and 39.8% (4093 people), respectively.

Supplementary Table 5. Prevalence rate of the subdomains KCL and FSI by frailty status

|  | Total (*n* = 10276) | | FSI×KCL^*^ | | | | | | | |  | FSI^†^ | | | |  | KCL^†^ | | | |
| --- | --- | --- | --- | --- | --- | --- | --- | --- | --- | --- | --- | --- | --- | --- | --- | --- | --- | --- | --- | --- |
|  |  |  | Non-frailty (*n* = 5960) | | Physical frailty (*n* = 223) | | Comprehensive frailty (*n* = 2211) | | Combinations (*n* = 1882) | |  | Non-frailty (*n* = 8171) | | Physical frailty (*n* = 2105) | |  | Non-frailty (*n* = 6183) | | Comprehensive frailty (*n* = 4093) | |
| **FSI** |  |  |  |  |  |  |  |  |  |  |  |  |  |  |  |  |  |  |  |  |
| Slow gait speed [*n* (%)] | 6649 | (64.7) | 2770 | (46.5) | 214 | (96.0) | 1809 | (81.8) | 1856 | (98.6) |  | 4579 | (56.0) | 2070 | (98.3) |  | 2984 | (48.3) | 3665 | (89.5) |
| Cognitive [*n* (%)] | 1234 | (12.0) | 281 | (4.7) | 79 | (35.4) | 121 | (5.5) | 753 | (40.0) |  | 402 | (4.9) | 832 | (39.5) |  | 360 | (5.8) | 874 | (21.4) |
| Exhaustion [*n* (%)] | 3374 | (32.8) | 597 | (10.0) | 166 | (74.4) | 909 | (41.1) | 1702 | (90.4) |  | 1506 | (18.4) | 1868 | (88.7) |  | 763 | (12.3) | 2611 | (63.8) |
| Low PA [*n* (%)] | 2187 | (21.3) | 630 | (10.6) | 135 | (60.5) | 362 | (16.4) | 1060 | (56.3) |  | 992 | (12.1) | 1195 | (56.8) |  | 765 | (12.4) | 1422 | (34.7) |
| Weight loss [*n* (%)] | 1663 | (16.2) | 387 | (6.5) | 104 | (46.6) | 179 | (8.1) | 993 | (52.8) |  | 566 | (6.9) | 1097 | (52.1) |  | 491 | (7.9) | 1172 | (28.6) |
| **KCL**^‡^ |  |  |  |  |  |  |  |  |  |  |  |  |  |  |  |  |  |  |  |  |
| IADL disability [*n* (%)] | 1716 | (16.7) | 0 | (0) | 0 | (0) | 615 | (27.8) | 1101 | (58.5) |  | 615.0 | (7.5) | 1101.0 | (52.3) |  | 0 | (0) | 1716 | (100) |
| Physical [*n* (%)] | 2933 | (28.5) | 349 | (5.9) | 19 | (8.5) | 1243 | (56.2) | 1322 | (70.2) |  | 1592.0 | (19.5) | 1341.0 | (63.7) |  | 368 | (6.0) | 2565 | (62.7) |
| Nutrition [*n* (%)] | 311 | (3.0) | 34 | (0.6) | 12 | (5.4) | 32 | (1.4) | 233 | (12.4) |  | 66.0 | (0.8) | 245.0 | (11.6) |  | 46 | (0.7) | 265 | (6.6) |
| Oral [*n* (%)] | 2592 | (25.2) | 496 | (8.3) | 20 | (9.0) | 963 | (43.6) | 1113 | (59.1) |  | 1459 | (17.9) | 1133 | (53.8) |  | 516 | (8.3) | 2076 | (50.7) |
| Social [*n* (%)] | 1125 | (10.9) | 137 | (2.3) | 5 | (2.2) | 393 | (17.8) | 590 | (31.3) |  | 530 | (6.5) | 595 | (28.3) |  | 142 | (2.3) | 983 | (24.0) |
| Cognitive [*n* (%)] | 4117 | (40.1) | 1323 | (22.2) | 50 | (22.4) | 1381 | (62.5) | 1363 | (72.4) |  | 2704 | (33.1) | 1413 | (67.1) |  | 1373 | (22.2) | 2744 | (67.0) |
| Depression [*n* (%)] | 3450 | (33.6) | 493 | (8.3) | 79 | (35.4) | 1213 | (54.9) | 1665 | (88.5) |  | 1706 | (20.9) | 1744 | (82.9) |  | 572 | (9.3) | 2878 | (70.3) |

FSI, frailty screening index; IADL, Instrumental Activities of Daily Living; KCL, Kihon Checklist; PA, physical activity

All value were shown in terms of the number of cases with percentage.

^*^ Four groups stratified by FSI and KCL.

^†^ Two groups stratified by FSI and KCL.

^‡^ The cutoff points of the KCL subdomains are the following: the cut-off point for IADL disability, physical inactivity, malnutrition, oral dysfunction, socialization domain, cognitive domain, and depression were defined as ≥10 points on 20 items, including shopping, ≥3 points on 5 items, including walk continuously and history of fall, 2 points on 2 items, including weight loss and low body mass index (<18.5 kg/m^2^), ≥2 points on 3 items, including dry mouth and poor mastication, ≥1 point on 2 items, including frequency of going out less, ≥1 point on 3 items, including memory loss, and ≥2 points on 5 items, including fulfillment and helpless, respectively.

Supplementary Table 6. Characteristics of the included and excluded participants ^*^

|  | Included participants (*n* = 10276) | | Excluded participants (*n* = 3018) | | *p*-value |
| --- | --- | --- | --- | --- | --- |
| Age [years] ^†^ | 73.9 | (6.8) | 76.5 | (6.9) | <0.001 |
| Women [*n* (%)] ^‡^ | 5580 | (54.3) | 1757 | (58.2) | <0.001 |
| PD ≥1000 people/km^2^ [*n* (%)] ^‡^ | 4633 | (45.1) | 1284 | (42.5) | 0.014 |
| Living alone [*n* (%)] ^‡^ | 1287 | (12.5) | 408 | (13.5) | 0.150 |
| HSES [*n* (%)] ^‡^ | 3339 | (32.5) | 889 | (29.5) | 0.002 |
| Education ≥13 y [*n* (%)] ^‡^ | 2103 | (20.5) | 464 | (15.4) | <0.001 |
| Current smoker [*n* (%)] ^‡^ | 1126 | (11.0) | 271 | (9.0) | 0.008 |
| Alcohol drinker [*n* (%)] ^‡^ | 6482 | (63.1) | 1795 | (59.5) | <0.001 |
| Sleep time [min] ^†^ | 412 | (94) | 411 | (94) | 0.813 |
| No medication [*n* (%)] ^‡^ | 2104 | (20.5) | 500 | (16.6) | <0.001 |
| Hypertension [*n* (%)] ^‡^ | 3894 | (37.9) | 1009 | (33.4) | <0.001 |
| Stroke [*n* (%)] ^‡^ | 476 | (4.6) | 141 | (4.7) | 0.927 |
| Heart disease [*n* (%)] ^‡^ | 1275 | (12.4) | 384 | (12.7) | 0.675 |
| Diabetes [*n* (%)] ^‡^ | 1108 | (10.8) | 282 | (9.3) | 0.023 |
| Hyperlipidemia [*n* (%)] ^‡^ | 924 | (9.0) | 198 | (6.6) | <0.001 |
| Digestive disease [*n* (%)] ^‡^ | 499 | (4.9) | 168 | (5.6) | 0.116 |
| Respiratory disease [*n* (%)] ^‡^ | 824 | (8.0) | 219 | (7.3) | 0.171 |
| Urological diseases [*n* (%)] ^‡^ | 646 | (6.3) | 171 | (5.7) | 0.212 |
| Cancer [*n* (%)] ^‡^ | 367 | (3.6) | 120 | (4.0) | 0.298 |
| No. of chronic diseases ^†,^^§^ | 0.9 | (1.0) | 1.0 | (1.0) | <0.001 |

HSES, high socioeconomic status; PD, population density

^*^ Data for participants with missing values were imputed by multiple imputation (*n* = [*n* in included participants] and [*n* in excluded participants]): family structure (*n* = 675 and 444); socioeconomic status (*n* = 395 and 335); education (*n* = 1139 and 756); smoking status (*n* = 257 and 445); alcohol status (*n* = 230 and 374); sleep time (*n* = 536 and 501); medications (*n* = 657 and 483).

^†^ Continuous variables were shown in terms of mean with standard deviation and were analyzed using variance analysis.

^‡^ Category variables were shown in terms of the number of cases with percentage and were analyzed using the Pearson's Chi-square test.

^§^ From the data obtained on disease status (including the presence of hypertension, stroke, heart disease, diabetes, hyperlipidemia, digestive disease, respiratory disease, urological diseases, and cancer), the individual scores were summed to obtain a total score ranging from 0 (no comorbidity) to 9 (poor status).

Supplementary Table 7. Results of sensitivity analysis for the relationship between frailty status and all-cause mortality after excluding participants with an event in the first two years of follow-up

|  | *n* | Event | PY | Event/1000 PY | | Model 1 ^*^ | | Model 2 ^†^ | |
| --- | --- | --- | --- | --- | --- | --- | --- | --- | --- |
|  |  |  |  | Rate | 95%CI | HR | 95%CI | HR | 95%CI |
| **FSI×KCL** |  | | | | | | | | |
| Non-frailty | 5873 | 259 | 30573 | 8.5 | (7.5 to 9.6) | 1.00 | (Ref) | 1.00 | (Ref) |
| Physical frailty | 218 | 9 | 1139 | 7.9 | (4.1 to 15.2) | 0.91 | (0.47 to 1.77) | 0.85 | (0.44 to 1.66) |
| Comprehensive frailty | 2120 | 276 | 10697 | 25.8 | (22.9 to 29.0) | 2.03 | (1.70 to 2.42) | 1.91 | (1.60 to 2.29) |
| Combinations | 1652 | 300 | 8134 | 36.88 | (32.9 to 41.3) | 2.44 | (2.03 to 2.93) | 2.21 | (1.83 to 2.67) |
| *Interaction* |  |  |  |  |  |  |  |  |  |
| RERI ^‡^ |  |  |  | 11.7 | (7.4 to 15.9) | 0.51 | (-0.25 to 1.26) | 0.46 | (-0.29 to 1.20) |
| RERI (%) |  |  |  | 41.0 | | 35.1 | | 36.7 | |
| **FSI** |  | | | | | | | | |
| Non-frailty | 7993 | 535 | 41270 | 13.0 | (11.9 to 14.1) | 1.00 | (Ref) | 1.00 | (Ref) |
| Physical frailty | 1870 | 309 | 9274 | 33.32 | (29.8 to 37.3) | 1.60 | (1.38 to 1.86) | 1.46 | (1.25 to 1.70) |
| **KCL** |  | | | | | | | | |
| Non-frailty | 6091 | 268 | 31712 | 8.5 | (7.5 to 9.5) | 1.00 | (Ref) | 1.00 | (Ref) |
| Comprehensive frailty | 3772 | 576 | 18832 | 30.6 | (28.2 to 33.2) | 2.21 | (1.89 to 2.59) | 2.05 | (1.74 to 2.41) |

CI, confidence interval; FSI, frailty screening index; HR, hazard ratio; KCL, Kihon Checklist; RERI, relative excess risk due to interaction; PY, person-years

^*^ Model 1: Adjusted for age, sex, and population density.

^†^ Model 2: In addition to the factors listed in Model 1, adjusted for family structure, economic status, educational attainment, smoking status, alcohol consumption status, sleep time, medication use, and number of chronic diseases.

^‡^ We estimated that p<0.05 when the 95% CI of the RERI exceeded 0, and p≥0.05 when the 95% CI of the RERI did not exceed 0.

Supplementary Table 8. Results of sensitivity analysis for the relationship between frailty status and all-cause mortality using complete case data

|  | *n* | Event | PY | Event/1000 PY | | Model 1 ^*^ | | Model 2 ^†^ | |
| --- | --- | --- | --- | --- | --- | --- | --- | --- | --- |
|  |  |  |  | Rate | 95%CI | HR | 95%CI | HR | 95%CI |
| **FSI×KCL** |  | | | | | | | | |
| Non-frailty | 4456 | 232 | 22987 | 10.1 | (8.9 to 11.5) | 1.00 | (Ref) | 1.00 | (Ref) |
| Physical frailty | 187 | 11 | 960 | 11.5 | (6.3 to 20.7) | 1.13 | (0.62 to 2.07) | 1.08 | (0.59 to 1.98) |
| Comprehensive frailty | 1554 | 259 | 7614 | 34.0 | (30.1 to 38.4) | 2.25 | (1.87 to 2.71) | 2.08 | (1.72 to 2.51) |
| Combinations | 1323 | 343 | 5972 | 57.4 | (51.7 to 63.8) | 3.36 | (2.80 to 4.04) | 2.96 | (2.45 to 3.57) |
| *Interaction* |  |  |  |  |  |  |  |  |  |
| RERI ^‡^ |  |  |  | 22.1 | (15.5 to 28.6) | 0.99 | (0.24 to 1.75) | 0.80 | (0.07 to 1.53) |
| RERI (%) |  |  |  | 46.6 | | 41.7 | | 40.7 | |
| **FSI** |  | | | | | | | | |
| Non-frailty | 6010 | 491 | 30602 | 16.0 | (14.7 to 17.5) | 1.00 | (Ref) | 1.00 | (Ref) |
| Physical frailty | 1510 | 354 | 6933 | 51.1 | (46.0 to 56.7) | 2.07 | (1.79 to 2.39) | 1.85 | (1.60 to 2.15) |
| **KCL** |  | | | | | | | | |
| Non-frailty | 4643 | 243 | 23947 | 10.1 | (8.9 to 11.5) | 1.00 | (Ref) | 1.00 | (Ref) |
| Comprehensive frailty | 2877 | 602 | 13587 | 44.3 | (40.9 to 48.0) | 2.71 | (2.30 to 3.18) | 2.43 | (2.06 to 2.88) |

CI, confidence interval; FSI, frailty screening index; HR, hazard ratio; KCL, Kihon Checklist; RERI, relative excess risk due to interaction; PY, person-years

^*^ Model 1: Adjusted for age, sex, and population density.

^†^ Model 2: In addition to the factors listed in Model 1, adjusted for family structure, economic status, educational attainment, smoking status, alcohol consumption status, sleep time, medication use, and number of chronic diseases.

^‡^ We estimated that p<0.05 when the 95% CI of the RERI exceeded 0, and p≥0.05 when the 95% CI of the RERI did not exceed 0.

Supplementary Table 9. Results of sensitivity analysis for the relationship between frailty status and all-cause mortality using frailty defined by ≥ 2 point of frailty screening index

|  | *n* | Event | PY | Event/1000 PY | | Model 1 ^*^ | | Model 2 ^†^ | |
| --- | --- | --- | --- | --- | --- | --- | --- | --- | --- |
|  |  |  |  | Rate | 95%CI | HR | 95%CI | HR | 95%CI |
| **FSI×KCL** |  | | | | | | | | |
| Non-frailty | 4961 | 282 | 25552 | 11.0 | (9.8 to 12.4) | 1.00 | (Ref) | 1.00 | (Ref) |
| Physical frailty | 1222 | 78 | 6271 | 12.4 | (10.0 to 15.5) | 1.18 | (0.91 to 1.51) | 1.17 | (0.91 to 1.50) |
| Comprehensive frailty | 926 | 145 | 4526 | 32.0 | (27.2 to 37.7) | 1.96 | (1.60 to 2.41) | 1.86 | (1.51 to 2.29) |
| Combinations | 3167 | 752 | 14635 | 51.4 | (47.8 to 55.2) | 2.80 | (2.41 to 3.24) | 2.66 | (2.28 to 3.09) |
| *Interaction* |  |  |  |  |  |  |  |  |  |
| RERI ^‡^ |  |  |  | 17.9 | (11.1 to 24.7) | 0.67 | (0.23 to 1.11) | 0.64 | (0.21 to 1.07) |
| RERI (%) |  |  |  | 55.7 | | 63.4 | | 62.0 | |
| **FSI** |  | | | | | | | | |
| Non-frailty | 5887 | 427 | 30078 | 14.2 | (12.9 to 15.6) | 1.00 | (Ref) | 1.00 | (Ref) |
| Physical frailty | 4389 | 830 | 20905 | 39.7 | (37.1 to 42.5) | 1.94 | (1.72 to 2.19) | 1.87 | (1.65 to 2.11) |

CI, confidence interval; FSI, frailty screening index; HR, hazard ratio; KCL, Kihon Checklist; RERI, relative excess risk due to interaction; PY, person-years

^*^ Model 1: Adjusted for age, sex, and population density.

^†^ Model 2: In addition to the factors listed in Model 1, adjusted for family structure, economic status, educational attainment, smoking status, alcohol consumption status, sleep time, medication use, and number of chronic diseases.

^‡^ We estimated that p<0.05 when the 95% CI of the RERI exceeded 0, and p≥0.05 when the 95% CI of the RERI did not exceed 0.

Supplementary Table 10. Hazard ratios for frailty status and all-cause mortality calculated using age-stratified multivariate Cox proportional hazards analysis

|  | *n* | Event | PY | Event/1000 PY | | Model 1 ^*^ | | Model 2 ^†^ | |
| --- | --- | --- | --- | --- | --- | --- | --- | --- | --- |
|  |  |  |  | Rate | 95%CI | HR | 95%CI | HR | 95%CI |
| ***<75 years*** |  |  |  |  |  |  |  |  |  |
| **FSI×KCL** |  | | | | | | | | |
| Non-frailty | 4,338 | 168 | 22484 | 7.5 | (6.4 to 8.7) | 1.00 | (Ref) | 1.00 | (Ref) |
| Physical frailty | 155 | 6 | 807 | 7.4 | (3.3 to 16.6) | 1.07 | (0.47 to 2.42) | 1.00 | (0.44 to 2.26) |
| Comprehensive frailty | 1,027 | 94 | 5201 | 18.1 | (14.8 to 22.1) | 2.36 | (1.84 to 3.05) | 2.18 | (1.68 to 2.82) |
| Combinations | 631 | 85 | 3038 | 28.0 | (22.6 to 34.6) | 3.59 | (2.76 to 4.66) | 2.99 | (2.26 to 3.95) |
| *Interaction* |  |  |  |  |  |  |  |  |  |
| RERI ^‡^ |  |  |  | 9.9 | (4.7 to 15.2) | 1.15 | (-0.05 to 2.36) | 0.81 | (-0.31 to 1.94) |
| RERI (%) |  |  |  | 48.5 | | 44.5 | | 40.9 | |
| **FSI** |  |  |  |  |  |  |  |  |  |
| Non-frailty | 5,365 | 262 | 27685 | 9.5 | (8.4 to 10.7) | 1.00 | (Ref) | 1.00 | (Ref) |
| Physical frailty | 786 | 91 | 3845 | 23.7 | (19.3 to 29.1) | 2.45 | (1.93 to 3.11) | 2.02 | (1.58 to 2.60) |
| **KCL** |  |  |  |  |  |  |  |  |  |
| Non-frailty | 4,493 | 174 | 23291 | 7.5 | (6.4 to 8.7) | 1.00 | (Ref) | 1.00 | (Ref) |
| Comprehensive frailty | 1,658 | 179 | 8239 | 21.7 | (18.8 to 25.2) | 2.81 | (2.28 to 3.47) | 2.48 | (1.99 to 3.09) |
| ***≥75 years*** |  |  |  |  |  |  |  |  |  |
| **FSI×KCL** |  | | | | | | | | |
| Non-frailty | 1,622 | 178 | 8193 | 21.7 | (18.8 to 25.2) | 1.00 | (Ref) | 1.00 | (Ref) |
| Physical frailty | 68 | 8 | 339 | 23.6 | (11.8 to 47.2) | 1.04 | (0.51 to 2.10) | 0.97 | (0.48 to 1.97) |
| Comprehensive frailty | 1,184 | 273 | 5592 | 48.8 | (43.4 to 55.0) | 1.84 | (1.51 to 2.23) | 1.74 | (1.43 to 2.12) |
| Combinations | 1,251 | 445 | 5330 | 83.5 | (76.1 to 91.6) | 2.91 | (2.42 to 3.50) | 2.68 | (2.22 to 3.25) |
| *Interaction* |  |  |  |  |  |  |  |  |  |
| RERI^‡^ |  |  |  | 32.8 | (22.2 to 43.4) | 1.04 | (0.26 to 1.82) | 0.98 | (0.20 to 1.75) |
| RERI (%) |  |  |  | 53.1 | | 54.3 | | 58.0 | |
| **FSI** |  |  |  |  |  |  |  |  |  |
| Non-frailty | 2,806 | 451 | 13785 | 32.7 | (29.8 to 35.9) | 1.00 | (Ref) | 1.00 | (Ref) |
| Physical frailty | 1,319 | 453 | 5669 | 79.9 | (72.9 to 87.6) | 1.97 | (1.72 to 2.26) | 1.84 | (1.60 to 2.12) |
| **KCL** |  |  |  |  |  |  |  |  |  |
| Non-frailty | 1,690 | 186 | 8532 | 21.8 | (18.9 to 25.2) | 1.00 | (Ref) | 1.00 | (Ref) |
| Comprehensive frailty | 2,435 | 718 | 10922 | 65.7 | (61.1 to 70.7) | 2.34 | (1.97 to 2.77) | 2.16 | (1.82 to 2.58) |

CI, confidence interval; FSI, frailty screening index; HR, hazard ratio; KCL, Kihon Checklist; RERI, relative excess risk due to interaction; PY, person-years

^*^ Model 1: Adjusted for age, sex, and population density.

^†^ Model 2: In addition to the factors listed in Model 1, adjusted for family structure, economic status, educational attainment, smoking status, alcohol consumption status, sleep time, medication use, and number of chronic diseases.

^‡^ We estimated that p<0.05 when the 95% CI of the RERI exceeded 0, and p≥0.05 when the 95% CI of the RERI did not exceed 0.

Supplementary Table 11. Hazard ratios for frailty status and all-cause mortality calculated using sex-stratified multivariate Cox proportional hazards analysis

|  | *n* | Event | PY | Event/1000 PY | | Model 1 ^*^ | | Model 2 ^†^ | |
| --- | --- | --- | --- | --- | --- | --- | --- | --- | --- |
|  |  |  |  | Rate | 95%CI | HR | 95%CI | HR | 95%CI |
| ***Women*** |  |  |  |  |  |  |  |  |  |
| **FSI×KCL** |  | | | | | | | | |
| Non-frailty | 3041 | 124 | 15770 | 7.9 | (6.6 to 9.4) | 1.00 | (Ref) | 1.00 | (Ref) |
| Physical frailty | 121 | 3 | 636 | 4.7 | (1.5 to 14.6) | 0.64 | (0.20 to 2.00) | 0.65 | (0.21 to 2.03) |
| Comprehensive frailty | 1289 | 163 | 6404 | 25.5 | (21.8 to 29.7) | 1.72 | (1.34 to 2.20) | 1.62 | (1.26 to 2.08) |
| Combinations | 1129 | 273 | 5153 | 53.0 | (47.1 to 59.7) | 2.76 | (2.17 to 3.51) | 2.52 | (1.97 to 3.23) |
| *Interaction* |  |  |  |  |  |  |  |  |  |
| RERI ^‡^ |  |  |  | 30.7 | (21.4 to 40.0) | 1.49 | (0.27 to 2.71) | 1.34 | (0.13 to 2.55) |
| RERI (%) |  |  |  | 68.0 | | 79.8 | | 82.7 | |
| **FSI** |  |  |  |  |  |  |  |  |  |
| Non-frailty | 4330 | 287 | 22173 | 12.9 | (11.5 to 14.5) | 1.00 | (Ref) | 1.00 | (Ref) |
| Physical frailty | 1250 | 276 | 5788 | 47.7 | (42.4 to 53.7) | 1.89 | (1.58 to 2.26) | 1.78 | (1.48 to 2.13) |
| **KCL** |  |  |  |  |  |  |  |  |  |
| Non-frailty | 3162 | 127 | 16405 | 9.1 | (6.5 to 9.2) | 1.00 | (Ref) | 1.00 | (Ref) |
| Comprehensive frailty | 2418 | 436 | 11556 | 36.0 | (34.3 to 41.4) | 2.19 | (1.76 to 2.74) | 2.01 | (1.61 to 2.52) |
| ***Men*** |  |  |  |  |  |  |  |  |  |
| **FSI×KCL** |  | | | | | | | | |
| Non-frailty | 2919 | 222 | 14908 | 14.9 | (13.1 to 17.0) | 1.00 | (Ref) | 1.00 | (Ref) |
| Physical frailty | 102 | 11 | 510 | 21.6 | (11.9 to 38.9) | 1.29 | (0.70 to 2.36) | 1.16 | (0.63 to 2.14) |
| Comprehensive frailty | 922 | 204 | 4389 | 46.5 | (40.5 to 53.3) | 2.23 | (1.83 to 2.71) | 2.09 | (1.71 to 2.55) |
| Combinations | 753 | 257 | 3216 | 79.9 | (70.7 to 90.3) | 3.37 | (2.79 to 4.07) | 2.98 | (2.44 to 3.63) |
| *Interaction* |  |  |  |  |  |  |  |  |  |
| RERI^‡^ |  |  |  | 26.8 | (17.6 to 36.0) | 0.89 | (0.10 to 1.67) | 0.74 | (-0.02 to 1.50) |
| RERI (%) |  |  |  | 41.2 | | 35.9 | | 36.7 | |
| **FSI** |  |  |  |  |  |  |  |  |  |
| Non-frailty | 3841 | 426 | 19296 | 22.1 | (20.1 to 24.3) | 1.00 | (Ref) | 1.00 | (Ref) |
| Physical frailty | 855 | 268 | 3726 | 71.9 | (63.8 to 81.1) | 2.22 | (1.89 to 2.60) | 1.95 | (1.65 to 2.29) |
| **KCL** |  |  |  |  |  |  |  |  |  |
| Non-frailty | 3021 | 233 | 15418 | 15.1 | (13.3 to 17.2) | 1.00 | (Ref) | 1.00 | (Ref) |
| Comprehensive frailty | 1675 | 461 | 7604 | 60.6 | (55.3 to 66.4) | 2.69 | (2.28 to 3.18) | 2.44 | (2.05 to 2.90) |

CI, confidence interval; FSI, frailty screening index; HR, hazard ratio; KCL, Kihon Checklist; RERI, relative excess risk due to interaction; PY, person-years

^*^ Model 1: Adjusted for age, sex, and population density.

^†^ Model 2: In addition to the factors listed in Model 1, adjusted for family structure, economic status, educational attainment, smoking status, alcohol consumption status, sleep time, medication use, and number of chronic diseases.

^‡^ We estimated that p<0.05 when the 95% CI of the RERI exceeded 0, and p≥0.05 when the 95% CI of the RERI did not exceed 0.

Supplementary Table 12. Multivariate Cox proportional hazards analysis for the subdomains KCL and FSI and all-cause mortality

|  | *n* | Event | PY | Event/1000 PY | | Model 1 ^*^ | | Model 2 ^†^ | |
| --- | --- | --- | --- | --- | --- | --- | --- | --- | --- |
|  |  |  |  | Rate | 95%CI | HR | 95%CI | HR | 95%CI |
| **FSI** |  |  |  |  |  |  |  |  |  |
| ***Slow gait speed*** |  | | | | | | | | |
| Non-case | 3627 | 225 | 18612 | 12.1 | (10.6 to 13.8) | 1.00 | (Ref) | 1.00 | (Ref) |
| Case | 6649 | 1032 | 32372 | 31.9 | (30.0 to 33.9) | 1.56 | (1.34 to 1.82) | 1.44 | (1.23 to 1.68) |
| ***Cognitive*** |  | | | | | | | | |
| Non-case | 9042 | 941 | 45331 | 20.8 | (19.5 to 22.1) | 1.00 | (Ref) | 1.00 | (Ref) |
| Case | 1234 | 316 | 5652 | 55.9 | (50.1 to 62.4) | 1.77 | (1.55 to 2.01) | 1.65 | (1.44 to 1.88) |
| ***Exhaustion*** |  | | | | | | | | |
| Non-case | 6902 | 624 | 34963 | 17.8 | (16.5 to 19.3) | 1.00 | (Ref) | 1.00 | (Ref) |
| Case | 3374 | 633 | 16020 | 39.5 | (36.6 to 42.7) | 1.64 | (1.46 to 1.83) | 1.53 | (1.36 to 1.72) |
| ***Low PA*** |  | | | | | | | | |
| Non-case | 8089 | 825 | 40694 | 20.3 | (18.9 to 21.7) | 1.00 | (Ref) | 1.00 | (Ref) |
| Case | 2187 | 432 | 10290 | 42.0 | (38.2 to 46.1) | 1.58 | (1.40 to 1.78) | 1.46 | (1.29 to 1.65) |
| ***Weight loss*** |  | | | | | | | | |
| Non-case | 8613 | 885 | 43297 | 20.4 | (19.1 to 21.8) | 1.00 | (Ref) | 1.00 | (Ref) |
| Case | 1663 | 372 | 7687 | 48.4 | (43.7 to 53.6) | 1.91 | (1.69 to 2.16) | 1.82 | (1.61 to 2.06) |
| **KCL** ^‡^ |  |  |  |  |  |  |  |  |  |
| ***IADL disability*** |  | | | | | | | | |
| Non-case | 8560 | 666 | 43595 | 15.3 | (14.2 to 16.5) | 1.00 | (Ref) | 1.00 | (Ref) |
| Case | 1716 | 591 | 7389 | 80.0 | (73.8 to 86.7) | 2.93 | (2.58 to 3.33) | 2.65 | (2.33 to 3.02) |
| ***Physical*** |  | | | | | | | | |
| Non-case | 7343 | 550 | 37482 | 14.7 | (13.5 to 16.0) | 1.00 | (Ref) | 1.00 | (Ref) |
| Case | 2933 | 707 | 13502 | 52.4 | (48.6 to 56.4) | 2.18 | (1.92 to 2.47) | 1.99 | (1.75 to 2.26) |
| ***Nutrition*** |  | | | | | | | | |
| Non-case | 9965 | 1134 | 49731 | 22.8 | (21.5 to 24.2) | 1.00 | (Ref) | 1.00 | (Ref) |
| Case | 311 | 123 | 1253 | 98.2 | (82.3 to 117.2) | 2.71 | (2.24 to 3.27) | 2.51 | (2.07 to 3.04) |
| ***Oral*** |  | | | | | | | | |
| Non-case | 7684 | 736 | 38739 | 19.0 | (17.7 to 20.4) | 1.00 | (Ref) | 1.00 | (Ref) |
| Case | 2592 | 521 | 12244 | 42.6 | (39.0 to 46.4) | 1.56 | (1.39 to 1.75) | 1.45 | (1.29 to 1.63) |
| ***Social*** |  | | | | | | | | |
| Non-case | 9151 | 925 | 46040 | 20.1 | (18.8 to 21.4) | 1.00 | (Ref) | 1.00 | (Ref) |
| Case | 1125 | 332 | 4944 | 67.2 | (60.3 to 74.8) | 1.93 | (1.69 to 2.21) | 1.85 | (1.61 to 2.12) |
| ***Cognitive*** |  | | | | | | | | |
| Non-case | 6159 | 493 | 31276 | 15.8 | (14.4 to 17.2) | 1.00 | (Ref) | 1.00 | (Ref) |
| Case | 4117 | 764 | 19708 | 38.8 | (36.1 to 41.6) | 1.72 | (1.53 to 1.93) | 1.59 | (1.41 to 1.79) |
| ***Depression*** |  | | | | | | | | |
| Non-case | 6826 | 549 | 34794 | 15.8 | (14.5 to 17.2) | 1.00 | (Ref) | 1.00 | (Ref) |
| Case | 3450 | 708 | 16190 | 43.7 | (40.6 to 47.1) | 1.92 | (1.71 to 2.16) | 1.79 | (1.58 to 2.01) |

CI, confidence interval; FSI, frailty screening index; HR, hazard ratio; IADL, Instrumental Activities of Daily Living; KCL, Kihon Checklist; PA, physical activity; PY, person-years

^*^ Model 1: Adjusted for age, sex, and population density.

^†^ Model 2: In addition to the factors listed in Model 1, adjusted for family structure, economic status, educational attainment, smoking status, alcohol consumption status, sleep time, medication use, and number of chronic diseases.

^‡^ The cutoff points of the KCL subdomains are the following: the cut-off point for IADL disability, physical inactivity, malnutrition, oral dysfunction, socialization domain, cognitive domain, and depression were defined as ≥10 points on 20 items, including shopping, ≥3 points on 5 items, including walk continuously and history of fall, 2 points on 2 items, including weight loss and low body mass index (<18.5 kg/m^2^), ≥2 points on 3 items, including dry mouth and poor mastication, ≥1 point on 2 items, including frequency of going out less, ≥1 point on 3 items, including memory loss, and ≥2 points on 5 items, including fulfillment and helpless, respectively.


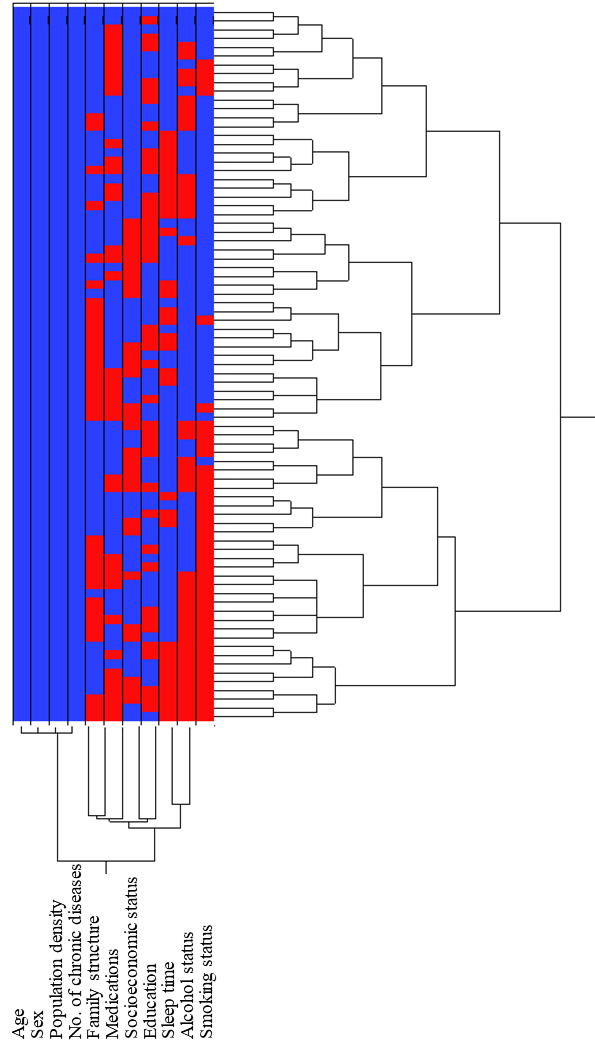


Supplementary Figure 1. Missing data pattern for covariates in this study

These covariates are included in multivariable analysis (blue for complete case and red for missing case).
